# Evaluation of ESAT6-CFP10 Skin Test for *Mycobacterium tuberculosis* Infection among Persons Living with HIV in China

**DOI:** 10.1128/jcm.01816-22

**Published:** 2023-03-22

**Authors:** Peng Lu, Kai Wu, Hongxi Zhou, Haibing Yu, Ju Yuan, Lang Dong, Qiao Liu, Xiaoyan Ding, Wei Lu, Haitao Yang, Limei Zhu, Leonardo Martinez

**Affiliations:** a Department of Chronic Communicable Disease, Jiangsu Provincial Center for Disease Control and Prevention, Nanjing, Jiangsu, People’s Republic of China; b Central Hospital, Jiangsu Prison Administration, Changzhou, Jiangsu, People’s Republic of China; c Jiangsu Provincial Health Development Research Center, Nanjing, Jiangsu, People’s Republic of China; d Department of Epidemiology, School of Public Health, Boston University, Boston, Massachusetts, USA; University of Manitoba

**Keywords:** tuberculosis, ESAT6-CFP10, tuberculosis infection, HIV, CD4, diagnostics

## Abstract

Recent global guidelines recommend Mycobacterium tuberculosis antigen-based skin tests, such as the ESAT6-CFP10 (EC) skin test, as acceptable alternatives to the tuberculin skin test (TST) and the QuantiFERON-TB Gold In-Tube test (QFT). However, the diagnostic value of these tests among persons living with HIV (PLHIV) is unknown. We aimed to assess the diagnostic accuracy of the EC among a cohort of PLHIV in China. We recruited PLHIV in Jiangsu Province, China, to assess sensitivity and specificity of the EC test. Participants were tested with the QFT, TST, and EC skin test. Results were stratified by age, M. tuberculosis BCG vaccination, and CD4 count. The sensitivity and specificity of the EC skin test was assessed using distinct cutoffs of the QFT and TST. Of 350 PLHIV enrolled in the study, 58 (16.6%), 89 (25.4%), and 59 (16.9%) tested positive with the EC test, the QFT, and the TST, respectively. Positivity increased with CD4 count; however, these trends were similar across tests. At a 5-mm cutoff, EC skin test specificity was high (99.6%, 95% confidence interval [CI] 95% CI = 97.7 to 100.0); however, sensitivity was moderate (81.4%; 95% CI = 66.6 to 91.6). After stratifying by BCG, the sensitivity and specificity were 86.4% (95% CI = 65.1 to 97.1) and 99.1% (95% CI = 95.0 to 100.0) among vaccinated PLHIV and 76.2% (95% CI = 52.8 to 91.8) and 100.0% (95% CI = 97.2 to 100.0) among unvaccinated PLHIV, respectively. Among PLHIV, the diagnostic value of the EC skin test remained high, regardless of BCG vaccination or CD4 count. The EC skin test performed comparably to TST and may be a valid alternative diagnostic test to use in settings or populations with high HIV prevalence and BCG vaccination. To our knowledge, this is the first study to evaluate the novel ESAT6-CFP10 skin test among PLHIV. Among 350 PLHIV, the test displayed high specificity and sensitivity, a finding which did not markedly differ based on BCG vaccination and CD4 count.

## INTRODUCTION

In 2019, more than 800,000 tuberculosis cases worldwide were linked to persons living with HIV (PLHIV), resulting in approximately 200,000 deaths (1). PLHIV are at multifold times higher risk to develop tuberculosis than individuals without HIV (2–4). HIV infection leads to an increased risk of progression to tuberculosis, even in the absence of low CD4^+^ T cell damage or when individuals are on antiretroviral therapy (5). For the purpose of reducing the risk of tuberculosis, preventive treatment is prioritized for PLHIV with recent Mycobacterium tuberculosis exposure or infection in national and international guidelines (6–9).

The tuberculin skin test (TST) and interferon gamma release assay (IGRA) are both recommended by World Health Organization (WHO) to diagnose M. tuberculosis infection (10). However, both of these tests have important limitations among PLHIV. PLHIV are often insensitive to TSTs, especially persons with CD4^+^ lymphocyte counts below 200 cells/mm^3^ (11, 12). The TST therefore may be an unreliable test for diagnosing M. tuberculosis in PLHIV. The sensitivity of IGRAs among PLHIV is debated. A systematic review and meta-analysis demonstrated that IGRAs performed similarly to the TST in identifying M. tuberculosis among PLHIV, suggestive insensitive results for IGRAs (13). In 2022, newer M. tuberculosis antigen-based skin tests were recommended as alternatives to diagnose M. tuberculosis infection (14). Among these tests is a novel ESAT6-CFP10 (EC) skin test (15). In recent studies, the EC skin test has shown similar diagnostic value to IGRAs among the general population (15), and a recent meta-analysis revealed M. tuberculosis antigen-based skin tests performed similarly to IGRA or TST for latent tuberculosis infection (16). However, to date, no data have been presented evaluating the diagnostic value of the EC skin test among PLHIV.

To address this question, we recruited PLHIV from a large prison in Jiangsu Province to assess the sensitivity and specificity of the EC skin test in this high-risk population. A diagnostic cutoff point was also determined to verify whether the cutoff criteria of general healthy participants (individuals without living with HIV) is similarly appropriate to PLHIV.

## MATERIALS AND METHODS

### Study population and design.

In 2021, we recruited incarcerated persons from a central prison hospital in Jiangsu Province, China. All incarcerated persons diagnosed with infectious diseases (including persons diagnosed with HIV, tuberculosis, gonorrhea, syphilis, and other infectious diseases) in the province are sent to this hospital for increased surveillance and health care. This study was performed during conventional check-ups for all provincial prisons, including routine blood testing, biochemical examination, hepatitis B serologic testing, syphilis antibody testing, hepatitis C antibody testing, electrocardiogram testing, chest X-ray film testing, CD4 count, and HIV load. Tuberculosis disease was excluded prior to participation in the study. Individuals refused to have EC skin test, TST and QFT were excluded. We did not exclude individuals with a low CD4^+^ lymphocyte count.

Among eligible persons, an EC test, a QFT, and a TST were used to evaluate M. tuberculosis infection among PLHIV. The EC antigen as a recombinant reagent, mainly including ESAT-6 and CFP-10, was developed by the Zhifei Longcom Biologic Pharmacy Company, China. The EC skin test was approved by the National Medical Products Administration as a standard single test diagnosing *M. tuberculosis* infection and is widely used in China.

### Procedures.

For eligible participants, sociodemographic information was collected through structured sociodemographic and clinical questionnaires by trained interviewers. We collected information on age, sex, height, weight, ethnicity, M. tuberculosis bacillus Calmette-Guérin (BCG) vaccination, and prison history. Chest X-ray films were obtained for all individuals prior to administration of the EC test, the TST, and the QFT to exclude tuberculosis disease. If the X-ray results were abnormal, a Gene Xpert test was used for tuberculosis diagnosis according to WHO guidelines (17). After tuberculosis was excluded, all participants were given each diagnostic test for M. tuberculosis infection. Blood samples were first collected for the QFT before administering the EC skin test and the TST (15). Individuals then received the TST on the volar surface of left forearm and the EC skin test on the right forearm. Both the TST and the EC skin test were performed using the Mantoux method (15). The TST and EC skin results were read 48 to 72 h after administration in accordance with the guidelines. TST responses were evaluated at cutoff points of 5 and 10 mm. The readers of the skin indurations were blind to the results of the QFT. Readers of the EC skin test were different from that those for the TST. They read the diameters of induration or redness independently without knowledge of the other reading. As part of routine care, CD4 cell counts were evaluated through blood sampling.

We used standard cutoffs for positivity of both the QFT and the TST. A positive QFT test was defined as a cutoff value that was ≥0.35 IU/mL. A positive TST result among PLHIV was defined as an induration reaction of ≥5 mm (18). We explored the use of other cutoffs for each test.

### Ethics approval and consent to participate.

This study was reviewed and approved by the ethics committee of Center for Disease Control and Prevention of Jiangsu Province. All eligible participants signed written informed consent. Prisoners with positive results from any of the three tests (TST/EC/QFT) could receive preventive treatments based on suggestions from physicians in the prison, but these were not compulsory.

### Statistical analysis.

We used 2 × 2 contingency tables in addition to means with standard deviations (SD) to summarize continuous and categorical variables. The Fisher exact test or a χ^2^ test was used to compare the three tests, as appropriate. Sensitivity, specificity, and overall diagnostic accuracy statistics were used to evaluate the concordance between the EC test and the TST or QFT. We also compared dichotomous QFT, TST, and EC skin test events by using Cohen’s kappa (κ) coefficient. Receiver operating characteristic (ROC) curves were used to identify cutoff values of the EC skin test using both the QFT and the TST as reference standards (individually and combinatorially). Cutoff values were determined by comparing ROC curves at distinct thresholds. Kappa coefficients were categorized as poor (κ ≤ 0.20), fair (0.20 < κ ≤ 0.40), moderate (0.40 < κ ≤ 0.60), good (0.60 < κ ≤ 0.80), and very good (0.80 < κ ≤ 1.00) (19).

Results were stratified by age, BCG vaccination, and CD4 count. The correlation between CD4 cell count and reaction diameter of the TST and the EC skin test and the level of gamma interferon (IFN-γ) was assessed using a Spearman correlation test. All data were analyzed using SPSS software (v23.0; IBM Corporation, Armonk, NY).

## RESULTS

### Demographic characteristics.

A total of 350 PLHIV were enrolled. Among them, 268 (76.6%) had a CD4 cell count of <500 cells/mm^3^. Most participants were male (89.7%) and of Han nationality (89.7%). 97 (27.9%) had previous incarceration history and the median incarceration time was 31.5 (interquartile range [IQR] = 13.0 to 55.8) months. Nearly half of participants were BCG vaccinated (Table 1). Among individuals with a CD4 count of <500 cells/mm^3^, 121 (45.5%) were BCG vaccinated, and among those with a CD4 count of ≥500 cells/mm^3^, 37 (45.1%) were BCG vaccinated. Seven (20%) individuals had indeterminate QFT results.

**TABLE 1:** Demographic characteristics of the included participants living with HIV in the study^*a*^

Characteristics	All	Participants with:	
		CD4 count, <500	CD4 count, ≥500
No. of participants	350	268	82
Median age, yrs (IQR)	38.0 (32.0–46.0)	38.0 (32.0–46.0)	37.0 (32.0–44.0)
Sex			
Male	314 (89.7)	244 (91.0)	70 (85.4)
Female	36 (10.3)	24 (9.0)	12 (14.6)
Median BMI (IQR)	22.2 (20.6–23.9)	22.0 (20.3–23.8)	22.8 (21.5–24.2)
Race			
Han	301 (89.7)	233 (87.6)	68 (82.9)
Minority	47 (10.3)	33 (12.4)	14 (17.1)
BCG vaccination			
No	190 (54.6)	145 (54.5)	45 (54.9)
Yes	158 (45.4)	121 (45.5)	37 (45.1)
Previous incarceration history			
No	251 (72.1)	190 (71.4)	61 (74.4)
Yes	97 (27.9)	76 (28.6)	21 (25.6)
Median incarceration time, mo (IQR)	31.5 (13.0–55.8)	29.0 (11.5–52.0)	41.0 (19.8–63.5)
Median EC, mm (IQR)	0.0 (0.0–1.0)	0.0 (0.0–1.0)	0.0 (0.0–1.0)
QFT			
Positive	89 (25.4)	67 (25.0)	22 (26.9)
Negative	254 (72.6)	195 (72.8)	59 (72.0)
Indeterminate	7 (0.0)	6 (2.2)	1 (1.2)
TST			
Positive	59 (16.9)	42 (15.7)	17 (20.7)
Negative	291 (83.1)	226 (84.3)	65 (79.3)
Median QFT (IQR)	0.01 (–0.01–0.97)	0.01 (0.00–0.59)	0.01 (–0.02–3.35)
Median TST (IQR)	0.0 (0.0–1.0)	0.0 (0.0–1.0)	0.0 (0.0–1.0)

a Data indicate medians with interquartile ranges (IQR) or the numbers (%). BMI, body mass index; EC, ESAT6-CFP10; TST, tuberculin skin test; QFT, QuantiFERON-TB Gold In-Tube test; BCG, bacillus Calmette-Guérin.

### Sensitivity and specificity of the EC skin test.

**FIG 1:**
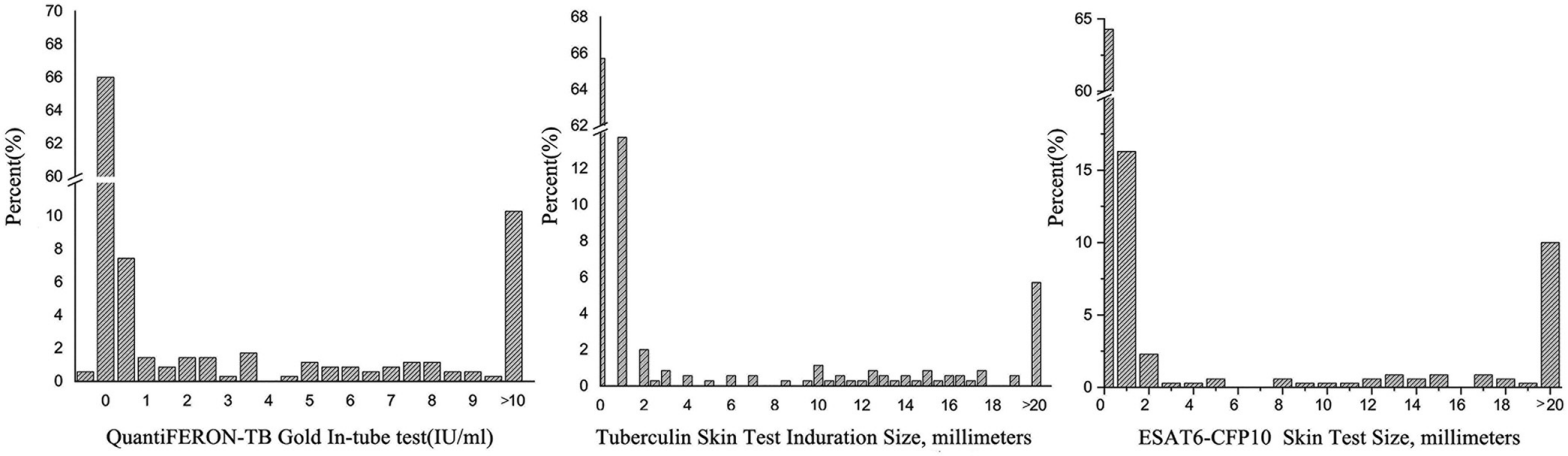
Distribution of QuantiFERON-TB Gold In-Tube test, tuberculin skin test, and ESAT6-CFP10 skin test.

**FIG 2:**
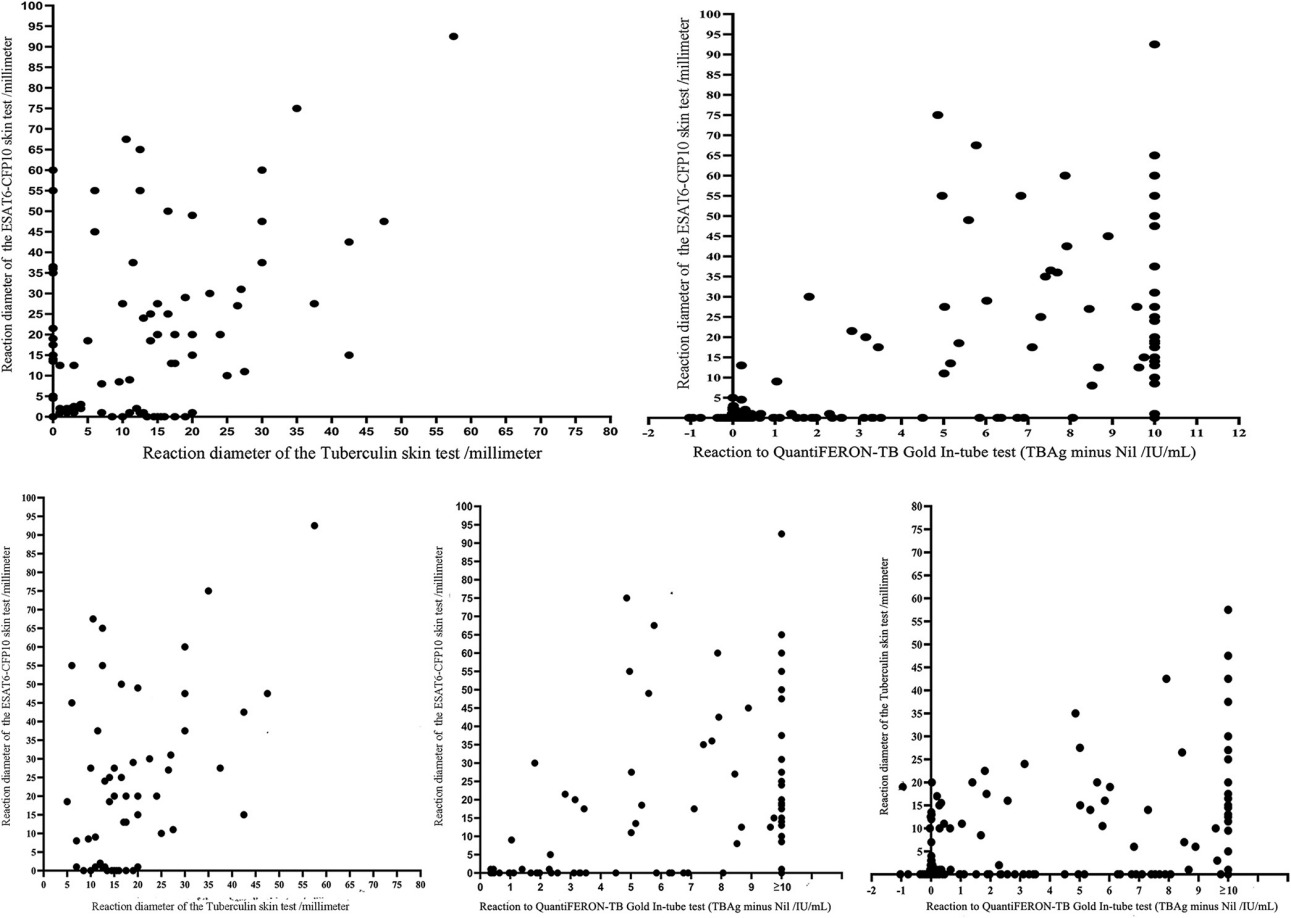
Scatterplots of the QuantiFERON-TB Gold In-Tube test, tuberculin skin test, and ESAT6-CFP10 skin test to describe correlation between tests.

**FIG 3:**
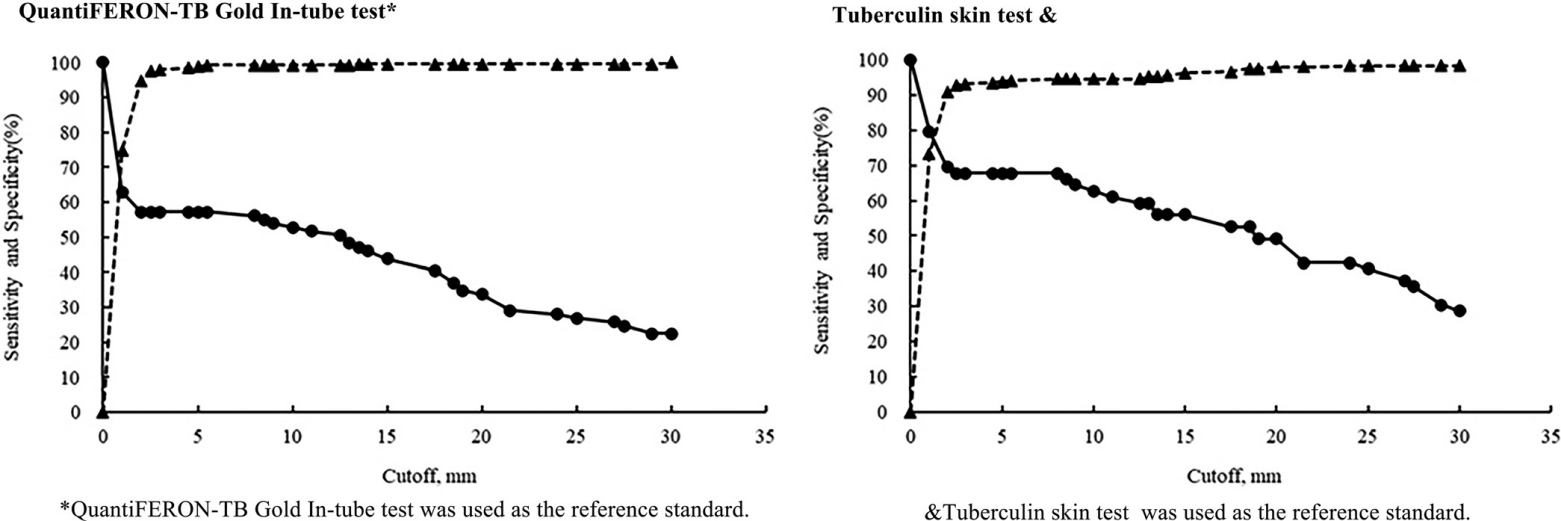
Sensitivity (circles) and specificity (triangles) of the ESAT6-CFP10 skin test with different cutoffs.

The mean EC response was 5.2 mm (SD, 13.5), higher than for the TST (3.4 mm; SD, 8.2) (*P = *0.001) (Fig. 1). EC responses correlated positively with the TST (Spearman rank, *r *= 0.651, *P < *0.001) (Fig. 2). The highest diagnostic values were achieved at a cutoff point of ≥5 mm when using as reference the QFT, TST, or both QFT and TST positive with sensitivities of 57.3% (95% CI = 46.4 to 67.7), 67.8% (95% CI = 54.4 to 79.4), and 81.4% (95% CI = 66.6 to 91.6) and specificities of 98.8% (95% CI = 96.6 to 99.8), 93.8 (95% CI = 90.4 to 96.3), and 99.6% (95% CI = 97.7 to 100.0) (Fig. 3; see also Fig. S1).

After stratifying by BCG using both the QFT and the TST as reference standards, the sensitivity and specificity were 86.4% (95% CI = 65.1 to 97.1) and 99.1% (95% CI = 95.0 to 100.0) among BCG-vaccinated PLHIV and 76.2% (95% CI = 52.8 to 91.8) and 100.0% (95% CI = 97.2 to 100.0) among non-BCG-vaccinated PLHIV, respectively (Table 2). The AUC values were 0.76 (95% CI = 0.71 to 0.80, *P < *0.001), 0.84 (95% CI = 0.80 to 0.88, *P < *0.001), and 0.91 (95% CI = 0.87 to 0.94, *P < *0.001), suggesting that ≥5mm may be the optimal cutoff value for the EC test (see Fig. S1 in the supplemental material). After we stratified the samples by BCG vaccination status, the highest diagnostic values were achieved at a cutoff point of ≥5 mm among persons with BCG (using the QFT and TST a combinatorially as a reference standard). The sensitivities and specificities at this cutoff were 65.0% (95% CI = 48.3 to 79.4) and 99.1% (95% CI = 95.3 to 100.0) and 70.0% (95% CI = 50.6 to 85.3) and 93.8% (95% CI = 88.1 to 97.3), respectively. However, among individuals not BCG vaccinated, the highest diagnostic values were achieved at a cutoff point of ≥4.5 mm for QFT as the reference standard, with a sensitivity and specificity of 50.0% (95% CI = 35.2 to 64.8) and 99.3% (95% CI = 96.0 to 100.0), and ≥1 mm for the TST as a reference standard, with a sensitivity and specificity of 69.0% (95% CI = 49.2 to 84.7) and 93.8% (95% CI = 88.9 to 97.0), respectively.

**TABLE 2:** Diagnostic performance of ESAT6-CFP10 skin test, QuantiFERON-TB Gold In-Tube test (≥0.35 IU/mL), and tuberculin skin test (≥5 mm)^*a*^

Parameter	EC skin test		QFT (≥0.35 IU/mL)		TST (≥5 mm)	
	*n/N*	Estimate (95% CI)	*n/N*	Estimate (95% CI)	*n/N*	Estimate (95% CI)
Sensitivity						
TST positive participants	40/59	67.8 (54.4–79.4)	43/56	76.8 (63.6–87.0)		
BCG vaccinated	21/30	70.0 (50.6–85.3)	22/29	75.9 (56.5–89.7)		
Unvaccinated	18/28	64.3 (44.1–81.4)	21/27	77.8 (57.7–91.4)		
QFT positive participants	51/89	57.3 (46.4–67.7)			43/89	48.3 (37.6–59.2)
BCG vaccinated	26/40	65.0 (48.3–79.4)			22/40	55.0 (38.5–70.7)
Unvaccinated	25/49	51.0 (36.3–65.6)			21/49	42.9 (28.8–57.8)
TST and QFT positive	35/43	81.4 (66.6–91.6)				
BCG vaccinated	19/22	86.4 (65.1–97.1)				
Unvaccinated	16/21	76.2 (52.8–91.8)				
Specificity						
TST negative participants	273/291	93.8 (90.4–96.3)	241/287	84.0 (78.0–87.0)		
BCG vaccinated	119/128	93.0 (87.1–96.7)	109/127	85.8 (78.5–91.4)		
Unvaccinated	153/162	94.4 (89.7–97.4)	131/159	82.4 (75.6–88.0)		
QFT negative participants	251/254	98.8 (96.6–99.8)			241/254	94.9 (91.4–97.2)
BCG vaccinated	114/116	98.3 (93.9–99.8)			109/116	94.0 (88.0–97.5)
Unvaccinated	136/137	99.3 (96.0–100.0)			131/137	95.6 (90.7–98.4)
TST and QFT negative	240/241	99.6 (97.7–100.0)				
BCG vaccinated	108/109	99.1 (95.0–100.0)				
Unvaccinated	131/131	100.0 (97.2–100.0)				

a EC, ESAT6-CFP10; TST, tuberculin skin test; QFT, QuantiFERON-TB Gold In-Tube test; *n/N*, number/total number; CI, confidence interval; BCG, bacillus Calmette-Guerin.

Among all PLHIV, 58 (16.6%) were EC skin test positive using 5 mm as the cutoff value. For the QFT and TST, 89 (25.4%) and 59 (16.9%) were positive, respectively. The proportion of EC test positivity was statistically similar to the TST positivity, but it was lower than the QFT positivity (*P = *0.002). Seven (<1%) participants had indeterminate QFT results. Of all participants with indeterminate results, 6 (85.7%) had a CD4^+^ cell count of <500 cells/mm^3^. Among participants with a CD4^+^ cell count of <500 cells/mm^3^, similar results were seen (*P = *0.003). However, among individuals with a CD4 cell count of ≥500 cells/mm^3^, there were no significant differences between EC test, QFT, and TST positivities (*P = *0.530). M. tuberculosis infection rates of PLHIV with a CD4 cell count of <500 cells/mm^3^ were lower than those with a CD4 cell count of ≥500 cells/mm^3^ for the EC skin test (15.3% versus 20.7%, *P = *0.247) and TST (15.7% versus 20.7%, *P = *0.284) but similar for the QFT (25.0% versus 26.9%, *P = *0.776). The positivity of the TST, QFT, and EC skin test increased with an increase of the CD4 cell count (see Fig. S2 in the supplemental material). We also found a strong correlation (*P < *0.001) between the CD4 cell count and the millimeter induration of the TST and EC skin test, as well as the quantitative level of IFN-γ. CD4^+^ levels predicting a positive QFT result were the same as for the EC skin test but much lower than for the TST (258 versus 480 cells/mm^3^, *P = *0.639).

The EC skin test showed higher sensitivity among individuals with a CD4 cell count of ≥500 cells/mm^3^ than that of individuals with a CD4 cell count of <500 cells/mm^3^ (100.0% versus 74.2%, *P = *0.0038). The specificities among TST- and QFT-negative participants were 93.8% (95% CI = 90.4 to 96.3) and 98.8% (95% CI = 96.6 to 99.8), respectively. The specificity of the EC skin test reached 99.6% (95% CI = 97.7 to 100.0) using a combinatorial outcome of TST- and QFT-negative results. The specificity of EC among individuals with a CD4 cell count of <500 cells/mm^3^ was lower than that of individuals with a CD4 cell count of ≥500 cells/mm^3^ (93.4% versus 95.4%, *P = *0.128) with negative TST; however, the result was the opposite among participants with a negative QFT (99.5% versus 96.6%, *P = *0.858) (see Fig. S3). The diagnostic performance of the EC skin test with a different cutoff value of QFT (≥0.7 IU/mL) and TST (≥10 mm) was similar (see Table S1).

### Diagnostic agreement of the EC skin test, TST, and QFT.

The diagnostic agreements between the EC skin test and the QFT, TST (≥5 mm), and TST (≥10 mm) were 88.0% (95% CI = 84.5 to 91.4), 83.1% (95% CI = 79.2 to 87.1), and 88.6% (95% CI = 85.2 to 91.9). The diagnostic agreement rate of PLHIV with a CD4 cell count of <500 cells/mm^3^ was less than that for PLHIV with a CD4 cell count of ≥500 cells/mm^3^ (88.2% versus 88.4% between the EC skin test and the QFT). However, between the EC skin test and TST, PLHIV with a CD4 cell count of <500 cells/mm^3^ had a lower diagnostic agreement rate (88.4% versus 92.7%, *P = *0.273 if TST was ≥5 mm and 87.3% versus 92.7%, *P = *0.181 if the TST was ≥10 mm) (Table 3). The diagnostic agreement of the EC skin test with a different cutoff value of QFT (≥0.7 IU/mL) and TST (≥10 mm) was similar (see Table S2).

**TABLE 3:** Agreement of diagnostic results for the EC skin test compared to the tuberculin skin test TST (≥5 mm) and the QuantiFERON-TB Gold In-Tube test (≥0.35 IU/mL)^*a*^

Participant category	EC skin test (≥5 mm)	QFT test (≥0.35 IU/mL)			TST (≥5 mm)		
		No. negative	No. positive	Consistency (95% CI)	No. negative	No. positive	Consistency (95% CI)
All participants	Negative	251	38	88 (84.5–91.4)	273	19	83.1 (79.2–87.1)
	Positive	3	51		40	18	
CD4 count, <500	Negative	194	30	88.2 (84.2–92.1)	211	16	88.4 (84.6–92.3)
	Positive	1	37		15	26	
CD4 count, ≥500	Negative	57	8	87.7 (80.3–95.0)	62	3	92.7 (86.9–98.4)
	Positive	2	14		3	14	

a EC, ESAT6-CFP10; TST, tuberculin skin test; QFT, QuantiFERON-TB Gold In-Tube test; CI, confidence interval.

## DISCUSSION

In our study, the diagnostic value, especially the specificity, of the EC skin test among PLHIV remained high. Importantly, this result remained consistent regardless of participant BCG vaccination status or CD4 count. Our results suggest 5 mm as a cutoff point for the EC skin test in our study population. The EC skin test still performed comparably to the TST after deleting some additional antigens not specific to M. tuberculosis. To our knowledge, our study is the first to evaluate the novel ESAT6-CFP10 skin test recently recommended by the WHO (15) among PLHIV and suggests that this test may represent a feasible alternative to traditionally used diagnostics.

IGRAs and TSTs are the most commonly used diagnostic tests to diagnose M. tuberculosis infection, including for PLHIV. Despite this, both tests have deficiencies among PLHIV since the sensitivity may be low for both TSTs and IGRAs (20). Furthermore, laboratory capacity must be available for the use of IGRAs (20). Our results suggest that the EC skin test may be a suitable alternative to the TST and IGRA similar to the recent meta-analysis (16). The specificity was high (>95%), while the sensitivity remained >80% when using a reference of TST and QFT positivity. Importantly, diagnostic markers remained relatively high among BCG-vaccinated (86%) and unvaccinated (76%) participants. BCG vaccination is now commonplace in China, but it is often used sparingly in PLHIV due to the high risk of disseminated BCG. Antigens used in conventional TST are not specific to M. tuberculosis and can be found in BCG and environmental nontuberculosis Mycobacterium spp., which would lead to high false positivity, resulting in unnecessary antibiotic treatment and potentially drug toxicity (15, 21, 22). After deletion of these additional antigens, the EC skin test still performed comparably to the TST, although less effectively than the QFT among individuals with a CD4 cell count of <500 cells/mm^3^. Furthermore, in view of the fact that PLHIV are frequently insensitive to skin testing, the EC skin test used the lager one of redness or induration in comparison with that TST used the induration of reaction (15). These results suggest that the EC skin test performed sufficiently regardless of vaccination status.

The optimal cutoff point found in our study was 5 mm from the EC skin test, which is broadly consistent with studies in the general population (23). However, unlike this study conducted in South Africa, the positivity of the EC skin test here was similar to that of the TST but lower than the QFT (23). The reasons for this observation remain unclear, but this incongruency may be due to HIV-associated immune impairment impacting the IGRA less than EC skin test and the TST (13). Considering the shorter incubation time with TST antigens, the degree of influence may more severe, leading to a higher proportion of nonresponders (24). IGRA performances might be less affected by HIV, possibly because the testing platform ensures that a sufficient number of mononuclear cells are available in the peripheral blood despite the overall low total CD4^+^ cells in whole blood (25, 26).

Similar to the TST, the sensitivity of the EC skin test among PLHIV was lower than the specificity. PLHIV are frequently insensitive to skin testing, especially persons with CD4^+^ lymphocyte counts below 100 cells/mm^3^ (11, 12). IGRA results were less likely to be positive in PLHIV, particularly for individuals with CD4^+^ lymphocyte counts below 300 cells/mm^3^ (27–29). In our study, compared to PLHIV with CD4^+^ lymphocyte counts higher than 500 cells/mm^3^, those with CD4^+^ lymphocyte counts below 500 cells/mm^3^ showed lower sensitivity and specificity. This was similar with C-Tb skin test that, which showed reduced sensitivities in those with CD4^+^ T cell counts below 100 cells/μL (23). The inverse correlation between TST, IGRA, and EC skin test positivity with the CD4^+^ lymphocyte count may be due to impairment in M. tuberculosis-specific CD4^+^ T cell function, along with consumption of a discrete subset of M. tuberculosis-specific IFN-γ^+^ IL-2^−^ TNF-α^+^ CD4 T cells. This would lead to CD4 T cell death after mycobacterial Ag stimulation of peripheral blood mononuclear cells from PLHIV and diminished M. tuberculosis-specific CD4 T cell proliferation (30).

The EC skin test has been widely used in China. Diagnostic value testing on M. tuberculosis through the EC skin test is limited among PLHIV, children and adolescents younger than 18 years, and household contacts (15, 20). Cy-Tb (Serum Institute of India) and Diaskintest (Generium, Russian Federation) are two other alternative newer M. tuberculosis antigen-based skin tests recommended by the WHO in recent guidance (20). Further head-to-head testing for these newer diagnostics would be useful for better understanding their value going forward. Five papers evaluating the Cy-Tb and Diaskintest among PLHIV found a pooled sensitivity of 63.5% (95% CI = 52.6 to 73.2%) (31–34). These studies included a total of 317 participants. This value is lower than our sensitivity estimates for the EC skin test in our study (81.4%). Comparing across studies here is difficult due to distinct study populations, reference standards, and BCG vaccination status conditions. A few studies have evaluated M. tuberculosis with the Cy-Tb and Diaskintest for other high-risk populations, such as children; in four studies in children, the combined pooled sensitivity of the Cy-Tb and Diaskintest was 97.1% (95% CI = 81.9 to 99.6%). However, the number of children included in these four studies was small (*n* = 34) (35–38). Although the data overall are limited, Guideline Development Group members supported extrapolation of the recommendation of Cy-Tb, Diaskintest, and the EC skin test to PLHIV and children and adolescents under 18 years old based on the available evidence. To fill in data and knowledge gaps for the EC skin test in these high-risk groups, our study may provide some elucidation; despite this, further data from diverse settings are needed.

There are limitations to this study. First, diagnosed tuberculosis cases are more commonly used to assess the sensitivity of diagnostic tests of M. tuberculosis infection (39). This may have led to an underestimation of the sensitivity of the EC skin test. Future studies should include an HIV-TB population to further determine the accuracy of the EC skin test among this population. Second, the classification of BCG vaccination was based on the BCG vaccine scar. Misclassification of the exposure can occur if a scar does not form. However, a BCG scar is a commonly used marker of vaccination since scar formation is a sensitive indicator of vaccination status (40–42). Furthermore, it is hard to distinguish false negatives from true negatives, particularly in PLHIV with low CD4 counts, such as for the gold standard of *M. tuberculosis* infection. What the IGRA does better than the TST is that the IGRA puts out an indeterminate result than a false negative. Lastly, incarcerated PLHIV may not be representative of the broader population of PLHIV. However, this population is important to the broader tuberculosis epidemic, and PLHIV are at highest risk of HIV in China, a setting with overall low prevalence (43, 44).

### Conclusions.

In conclusion, the diagnostic value of the EC skin test remains high among PLHIV, regardless of BCG vaccination status or CD4 count. This is especially apparent for diagnostic specificity. A cutoff point of 5 mm was found to be optimal for the EC skin test among this PLHIV cohort. The EC skin test still performs comparably to the TST after deletion of some additional antigens not specific to M. tuberculosis. In light of the deficiencies of current diagnostic tools, including laboratory requirements, indeterminate results, and high cost, the EC skin test may offer a reasonable alternative for diagnosing M. tuberculosis infection among PLHIV.
