# Supplementary material for: Evaluation of ESAT6-CFP10 Skin Test for Mycobacterium tuberculosis Infection among Persons Living with HIV in China
Source: J Clin Microbiol. 2023 Mar 22;61(4):e01816-22. doi: 10.1128/jcm.01816-22 (PMC10117090; doi:10.1128/jcm.01816-22)
Supplement: Supplemental file 1 — Supplemental material. Download jcm.01816-22-s0001.pdf, PDF file, 4.5 MB [file jcm.01816-22-s0001.pdf]

Appendix Tables and Appendix Figures

Appendix Table 1. Diagnostic performance of ESAT6-CFP10 skin test, QuantiFERON-TB Gold In-tube test(≥0.7 IU/mL), and tuberculin skin test(≥10mm).

Appendix Table 2. Agreement of the diagnostic results of EC skin test compared with tuberculin skin test(≥10mm) and QuantiFERON-TB Gold In-tube test(≥0.7 IU/mL).

Appendix Figure 1. Receiver operating curve analysis of ESAT6-CFP10 skin test for diagnosis of latent tuberculosis infection.

Appendix Figure 2. Positive rates of tuberculin skin test, QuantiFERON Gold In-Tube and ESAT6-CFP10 skin test according to CD4 cell count.

Appendix Figure 3. Diagnostic performance of ESAT6-CFP10 skin test, QuantiFERON-TB Gold In-tube test(≥0.35 IU/mL), and tuberculin skin test(≥5mm) stratified by CD4 count.

Appendix Table 1. Diagnostic performance of ESAT6-CFP10 skin test, QuantiFERON-TB Gold In-tube test(≥0.7 IU/mL), and tuberculin skin test(≥10mm).

**Test performance**	**EC skin test**		**QFT test (≥0.7IU/mL)**		**TST (≥10mm)**	
	**n/N**	**Estimate (95% CI)**	**n/N**	**Estimate (95% CI)**	**n/N**	**Estimate (95% CI)**
**Sensitivity**						
TST positive participants	40/59	67.8 (54.4, 79.4)	41/56	73.2 (59.7, 84.2)	…	…
CD4 Count <500	26/42	61.9 (45.6, 76.4)	29/39	74.4 (57.9, 87.0)	…	…
CD4 Count ≥500	14/17	82.4 (56.6, 96.2)	12/17	70.6 (44.0, 89.7)	…	…
QFT positive participants	51/89	57.3 (46.4, 67.7)	…	…	38/89	42.7 (32.3, 53.6)
CD4 Count <500	37/67	55.2 (42.6, 67.4)	…	…	27/67	40.3 (28.5, 53.0)
CD4 Count ≥500	14/22	63.6 (40.7, 82.8)	…	…	11/22	50.0 (28.2, 71.8)
TST and QFT positive	35/43	81.4 (66.6, 91.6)	…	…	…	…
CD4 Count <500	23/31	74.2 (55.4, 88.1)	…	…	…	…
CD4 Count ≥500	12/12	100.0 (73.5, 100.0)	…	…	…	…
**Specificity**						
TST negative participants	273/291	93.8 (90.4, 96.3)	245/287	85.4 (80.7, 89.2)	…	…
CD4 Count <500	211/226	93.4 (89.3, 96.2)	189/223	84.8 (79.4, 89.2)	…	…
CD4 Count ≥500	62/65	95.4 (87.1, 99.0)	56/64	87.5 (76.8, 94.4)	…	…
QFT negative participants	251/254	98.8 (96.6, 99.8)	…	…	242/254	95.3 (91.9, 97.5)
CD4 Count <500	194/195	99.5 (97.2, 100.0)	…	…	187/195	95.9 (92.1, 98.2)
CD4 Count ≥500	57/59	96.6 (88.3, 99.6)	…	…	55/59	93.2 (83.5, 98.1)
TST and QFT negative	240/241	99.6 (97.7, 100.0)	…	…	…	…
CD4 Count <500	186/187	99.5 (97.1, 100.0)	…	…	…	…
CD4 Count ≥500	54/54	100.0 (93.4, 100.0)	…	…	…	…

EC = ESAT6-CFP10. TST = tuberculin skin test. QFT=QuantiFERON-TB Gold In-tube test. CI = Confidence interval.

Appendix Table 2. Agreement of the diagnostic results of EC skin test compared with tuberculin skin test(≥10mm) and QuantiFERON-TB Gold In-tube test(≥0.7 IU/mL).

Participants	EC skin test (≥5mm)	QFT test(≥0.7 IU/mL)			TST(≥10mm)		
		Negative n	Positive n	Consistency (95% CI)	Negative n	Positive n	Consistency (95% CI)
All participants	Negative	257	32	89.8	275	17	88.6
	Positive	3	51	(86.6, 93.0)	23	35	(85.2, 91.9)
CD4 count <500	Negative	198	26	89.7	212	15	87.3
	Positive	1	37	(86.0, 93.4)	19	22	(83.3, 91.3)
CD4 count ≥500	Negative	59	6	90.1	63	2	92.7
	Positive	2	14	(83.5, 96.8)	4	13	(86.9, 98.4)

EC = ESAT6-CFP10. TST = tuberculin skin test. QFT=QuantiFERON-TB Gold In-tube test. CI = Confidence interval.

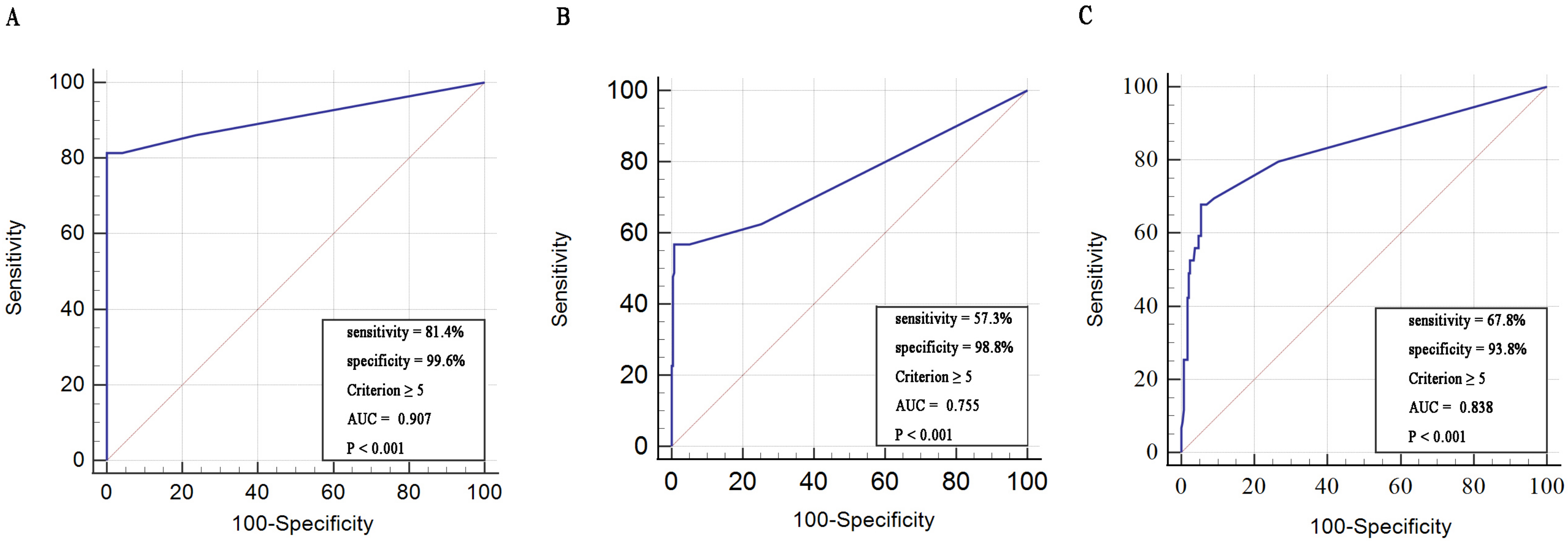

Note: A: tuberculin skin test (≥5mm) and QuantiFERON-TB Gold In-tube test(≥0.35 IU/mL) were used as the reference standard;

B.QuantiFERON-TB Gold In-tube test(≥0.35 IU/mL) was used as the reference standard; C: tuberculin skin test(≥5mm) used was used as the reference standard.

The blue line is the diagonal reference line, which represents the area under curve is 0.5.

The red line is the receiver operating characteristic curve composed of sensitivity and specificity of each point.

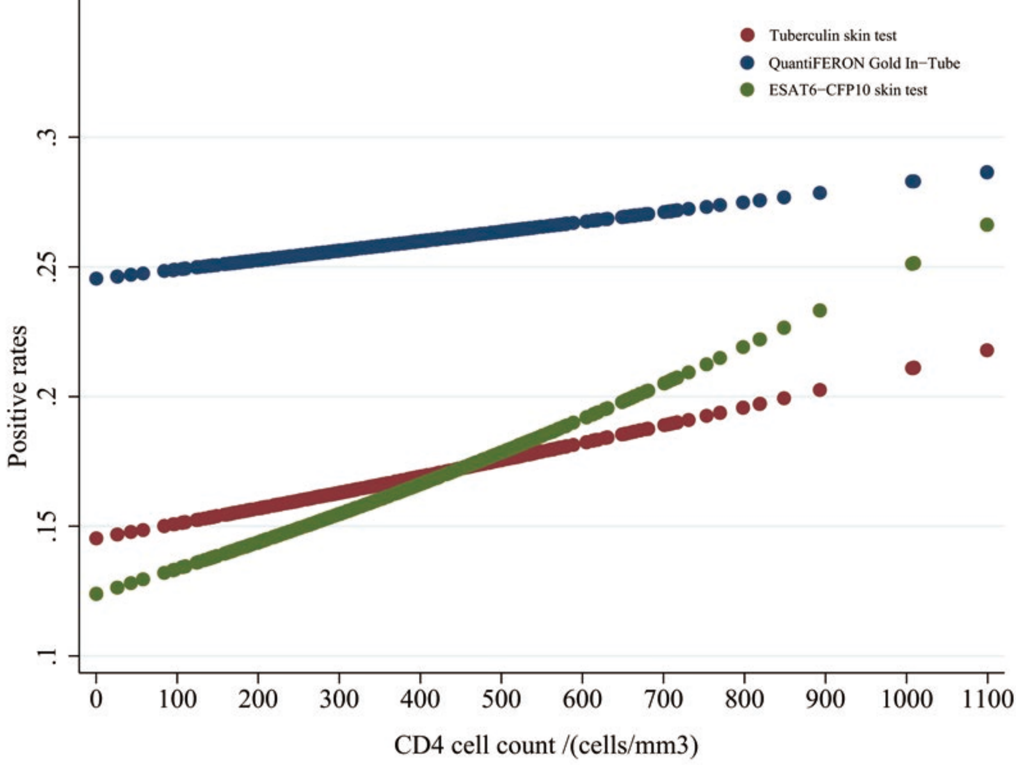
Tuberculin skin test
QuantiFERON Gold In−Tube
ESAT6−CFP10 skin test
Positive rates
.1
.15
.2
.25
.3
0
100
200
300
400
500
600
700
800
900
1000
1100
CD4 cell count /(cells/mm3)

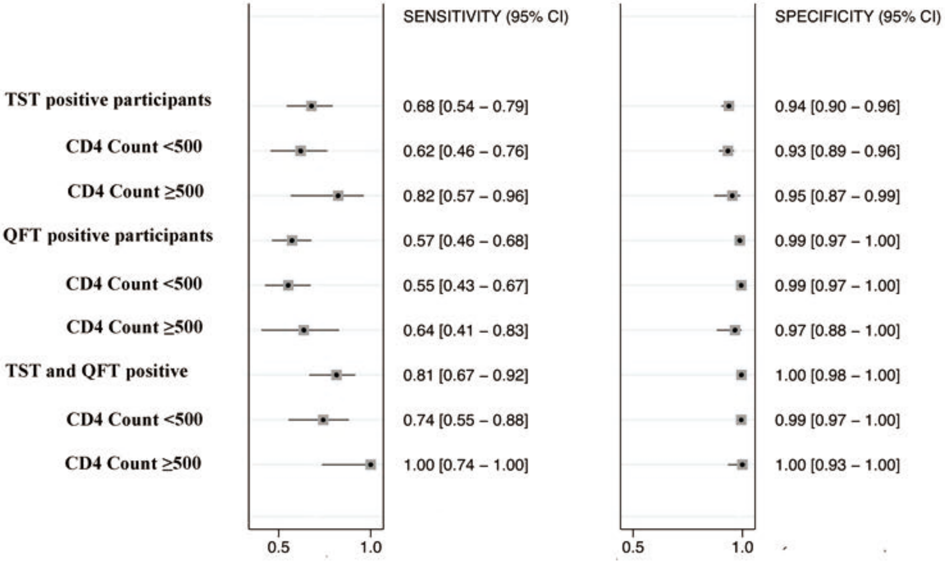

Note: QFT and TST were used as the reference standard.

TST = tuberculin skin test. QFT=QuantiFERON-TB Gold In-tube test.
